# Dynamic flow and shear stress as key parameters for intestinal cells morphology and polarization in an organ-on-a-chip model

**DOI:** 10.1007/s10544-021-00591-y

**Published:** 2021-10-16

**Authors:** Chiara A. M. Fois, Aaron Schindeler, Peter Valtchev, Fariba Dehghani

**Affiliations:** 1 School of Chemical and Biomolecular Engineering, The University of Sydney, Sydney, 2006 Australia; 2 Centre for Advanced Food Engineering, The University of Sydney, Sydney, 2006 Australia; 3 Bioengineering & Molecular Medicine Laboratory, The Children’s Hospital at Westmead and the Westmead Institute for Medical Research, Westmead, 2145 Australia

**Keywords:** Organ-on-a-chip, Microfluidics, Cell differentiation, Intestinal models, Computational fluid dynamics

## Abstract

Gut-on-a-chip microfluidic devices have emerged as versatile and practical systems for modeling the human intestine *in vitro*. Cells cultured under microfluidic conditions experience the effect of shear stress, used as a biomechanical cue to promote a faster cell polarization in Caco-2 cells when compared with static culture conditions. However, published systems to date have utilized a constant flow rate that fails to account for changes in cell shear stress (${\tau }_{c}$) resulting from changes in cell elongation that occur with differentiation. In this study, computational fluid dynamics (CFD) simulations predict that cells with villi-like morphology experience a ${\tau }_{c}$ higher than bulge-like cells at the initial growth stages. Therefore, we investigated the use of a dynamic flow rate to maintain a constant ${\tau }_{c}$ across the experiment. Microscopic assessment of cell morphology and dome formation confirmed the initiation of Caco-2 polarization within three days. Next, adopting our dynamic approach, we evaluated whether the following decreased flow could still contribute to complete cell differentiation if compared with the standard constant flow methodology. Caco-2 cells polarized under both conditions, secreted mucin-2 and villin and formed tight junctions and crypt-villi structures. Gene expression was not impacted using the dynamic flow rate. In conclusion, our dynamic flow approach still facilitates cell differentiation while enabling a reduced consumption of reagents.

**Supplementary information:**

The online version contains supplementary material available at 10.1007/s10544-021-00591-y.

## Introduction

In the development of functional foods, pharmaceuticals, and nutraceuticals it is important to understand their absorption in the gastrointestinal tract (GIT). Different approaches are used to estimate the absorption of active compounds in the GIT such as in silico simulations, *in vitro* models, *in vivo* testing on animal models, and clinical trials (Fois et al. 2019). Clinical trials are the gold standard for measuring the absorption of active compounds and their functionality, yet they are neither a viable nor cost-effective approach for screening libraries of bioactive compounds. Animal models can be used for screening candidate compounds, but they are poorly suited for high-throughput screening and do not always fully translate to human responses (Nguyen et al. 2015; Hugenholtz and de Vos 2018; Pound and Ritskes-Hoitinga 2018). Emerging microfluidic systems, known as organs-on-chips, have been developed in recent years as versatile and advanced culture systems for modelling normal cellular physiology and diseases. They have been applied to a variety of cell lineages to mimic more closely different human organs and their crosstalk. With the use of microfluidics conditions, the cells cultured in these micro-size chambers experience physiological conditions similar to those naturally occurring *in vivo* in the human body, a feature that makes the organs-on-chips a competitive new *in vitro* testing tool for product development and for screening the efficacy and absorption of drugs and active compounds (Ingber 2020; Van Den Berg et al. 2019).

Various gut-on-a-chip have been developed during the last decade, incorporating different cell types. These range from patient-derived cells to other immortalized cell lines with different features of the human intestine (Beaurivage et al. 2020; Gijzen et al. 2020; Kasendra et al. 2018; Jalili-Firoozinezhad et al. 2019). Colorectal carcinoma Caco-2 cells are widely used for studying the absorption and efficacy of active compounds in both standard and the microfluidic gut-on-a-chip systems* in vitro*. One of the important features offered by Caco-2 cells is their spontaneous differentiation into column-shaped enterocytes that resembles the morphology of intestinal villi (Hidalgo et al. 1989). When cultured in standard plates, cell differentiation and polarization is commonly completed within 21 days (Natoli et al. 2012; Lea 2015). Early studies trialed long-term cultures (16–31 days) of Caco-2 cells using gut-on-a-chip devices (Kimura et al. 2008; Mahler et al. 2009). However, it was revealed that Caco-2 cells differentiate up to three times faster in microfluidic devices compared to standard culture systems (Pocock et al. 2017). The 3D epithelial structures can be formed within 3 days of cultures in a microfluidic gut-on-a-chip (Chi et al. 2015) with or without the addition of mechanical cyclic strain (Kim et al. 2012; Kim and Ingber 2013). The combination of reduced size and more rapid cellular differentiation reduces the consumption of cells and reagents. The faster cell polarization is attributed to shear stress, which resembles the physiological conditions seen *in vivo*.

For the specific case of gut-on-a-chip models, usually, a constant flow rate is used across the cultured cell monolayer. The current practice is to aim to achieve the optimal shear stress of 0.02 dyne/cm^2^, previously proven to promote cell polarization. However, Caco-2 cells change morphology and dimensions during cell polarization and the resulting changes in cell shear stress (${\tau }_{c}$) during this process have not been thoroughly investigated. Shim and others reported that the microvilli observed on Caco-2 cells grown on a 3D scaffold were more damaged than those of cells grown on 2D monolayers under the same constant flow rate (Shim et al. 2017). The authors related this difference to the increased shear stress experienced by cells grown on scaffolds, which reasons our hypothesis that a constant flow rate may not always be the optimal solution for cells changing their morphologies. Likewise, as shown by Chi and others (Chi et al. 2015), Caco-2 cells polarization could only be promoted in their microfluidic system at a minimal flow rate of 0.5 µl/min and shear stress of 0.02 dyne/cm^2^.

Researchers often apply the *bulge model* theory (Gaver III et al. 1998), which allows determining cell shear stress (${\tau }_{c}$) by approximating cells as bulges. We speculate that this may not be the optimal approximation for polarized Caco-2 cells as they assume a columnar shape during their differentiation.

In our study, the effect of decreasing shear stress on Caco-2 cells in a gut-on-a-chip model was investigated to account for cell morphology changes. Therefore, we adopted a dynamic flow rate during cell growth. Additionally, a dynamic flow rate approach can be particularly important to further reduce the volumes and costs involved, such as in the case of expensive compounds in drug development research, where the gut-on-a-chip models can be adopted. It was hypothesized that decreasing the flow rate after the initial stages of cell growth, may have a beneficial effect on villi formation as a result of reducing the shear stress. To this end, computational fluid dynamics (CFD) simulations were used to determine the shear stress on cells of different dimensions. Specifically, we calculated which flow rates could maintain ${\tau }_{c}$ matching that initially experienced by cells at the early stages of cell growth and polarization. The dynamic flow rate conditions were then applied *in vitro,* and droplet digital PCR was performed to quantify any differences in gene expressions. Scanning electron and confocal microscopy were used to examine cell morphology, microvilli formation and crypt-villi structures, to investigate cell polarization. Furthermore, immunofluorescence staining was used to investigate the expression of classic differentiation markers, such as mucin and villin, and to measure cell monolayer’s heights. The establishment of tight junctions was also confirmed via immunostaining to verify the morphological integrity of the intestinal barrier established *in vitro*.

## Materials and methods

### Gut-on-a-chip fabrication

**Fig. 1:**
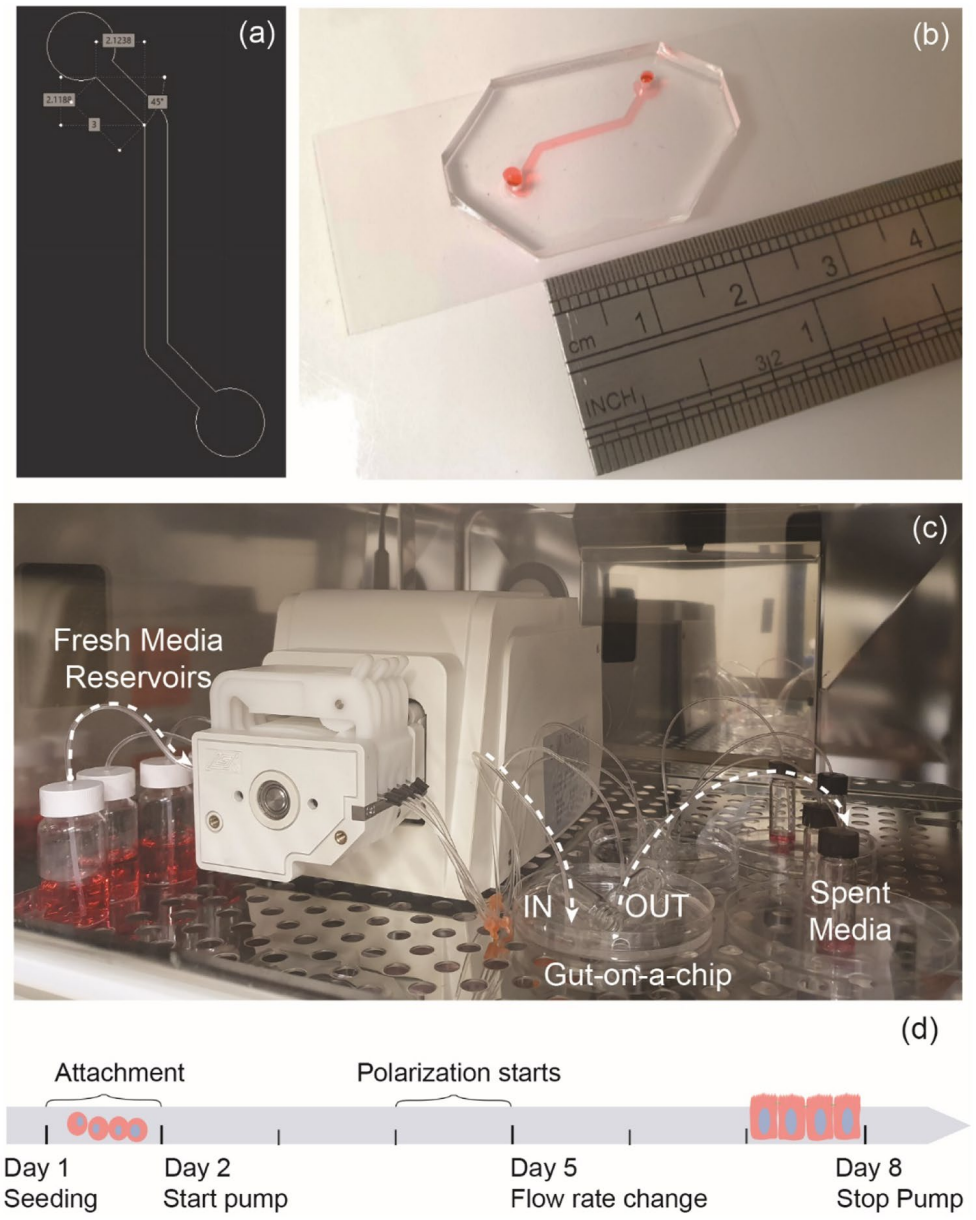
(**a**) Design in AutoCAD (Autodesk) of the gut-on-a-chip with dimensions in millimeters. (**b**) Photograph of the fabricated device and (**c**) of the setup inside the cell culture incubator. (**d**) Experimental design with time points for the flow rate variation study

The gut-on-a-chip device was designed on AutoCAD (Autodesk) as shown in Fig. 1(a) and consisted of a simple single chamber device with an inlet and outlet of 3 mm diameter. The channel’s width and length (excluding inlet and outlet) were respectively 1 mm and 12 mm. The gut-on-a-chip was fabricated with soft lithography techniques. First, SU-8 2050 (MicroChem) photoresist was spin-coated on a silicon wafer and the coated wafer was then exposed to UV on an EVG® 610 mask alignment system together with a soda-lime glass photomask containing the design. Following the post-exposure bake (5 min at 65 °C, then 15 min at 95 °C), the wafer was finally swirled in propylene glycol monomethyl ether acetate (MicroChem) for design development. The final height of the fabricated channel on the silicon wafer substrate was 150 µm. To fabricate the gut-on-a-chip, polydimethylsiloxane (PDMS) (Sylgard^TM^184 Dow Corning) was prepared in a ratio w/w 10:1 of PDMS to crosslinking agent, crosslinking agent, mixed and degassed in a vacuum desiccator to remove any bubbles. The solution was then cast on the silicon wafer and baked for 24 h at 60 °C. The PDMS chips were then cut to shape and inlets and outlets of 1.5 mm were created with biopsy punchers. Finally, the chips were bonded to glass coverslips (Menzel-Glazer #1, 24 × 60 mm) to enclose the channels after plasma treatment of the PDMS and glass surfaces (Harrick Plasma, PDC-002-HP, 230 V). A photograph of the device is shown in Fig. 1(b).

### Cell culture in the gut-on-a-chip

Caco-2 HTB-37™ intestinal cells were harvested in flasks at a cell seeding of 3 × 10^4^ cells/cm^2^. Cells were maintained in Dulbecco’s Modified Eagle Medium (DMEM Gibco™ 11,885,084) supplemented with 10% Fetal Bovine Serum (FBS, Sigma, F2442), 1% Antibiotic–Antimycotic (Gibco™ 15,240–062), and 1% Glutamax™ (Gibco™ 35,050–061). All experiments were performed with cells passages between 30 and 50. For experiments in the gut-on-a-chip, the devices were sterilized with 70% ethanol and exposed to UV light for one hour. The chips were then coated with Matrigel extracellular matrix (Corning Life Sciences, 356,231) at a final concentration (1:30) in serum-free F12 media (Gibco™ 11,320,033) and incubated at 37 °C for 1 h. Cells were trypsinized and manually seeded in each chip or multiwell plates at a density of 2 × 10^5^ cells/cm^2^. The growth area of the microfluidic channel shown in Fig. 1(b) was 0.32 cm^2^, resulting in a total number of 6.4 × 10^4^ cells per chip. Cells were left to adhere to the chips overnight.

### Computational fluid dynamics simulations

Computational Fluid Dynamics (CFD) simulations were run with Ansys Fluent (version 19.3) to investigate the effect of flow rate on the cell shear stress at different morphologies. Simulations were performed for the laminar flow of a Newtonian fluid with the same characteristics as the DMEM media used experimentally and supplemented with 10% FBS as previously observed by Poon (Poon 2020). Hence, the density $\rho$ and dynamic viscosity $\mu$, characteristic of the fluid, were assumed constant and taken respectively as expressed by Eqs. (1) and (2):

1 $$\rho =1 \mathrm{g}/{\mathrm{cm}}^{3}$$

2 $$\mu =9.3\times {10}^{-8} \mathrm{N}.\mathrm{s}/ {\mathrm{cm}}^{2}$$

No interactions with the side or top walls were considered as these are located far from the cells, and the walls were considered as being non-permeable. A zero-slip condition was applied, and the shear stress at the wall of the microchannel $\left({\tau }_{w}\right)$ was described after the parallel-plate model (Van Kooten et al. 1992), for which ${\tau }_{w}$ under laminar and uniform flow is a function of the volumetric flow rate ($Q$), dynamic viscosity ($\mu$) and the height ($h$) and width ($w$) of the microchannel. A shear stress value of 0.02 dyne/cm^2^ was used to determine the empty channel flow rate. This value is used in several studies for inducing Caco-2 cells polarization in gut-on-a-chip models (Kim et al. 2012; Pocock et al. 2017). By imposing the fluid properties from Eq. (2) and shear stress, the flow rate was calculated by Eq. (3).

3 $$Q=\frac{{\tau }_{w} w{h}^{2}}{6\mu }=29 \mu \mathrm{L}/\mathrm{hr}\cong 0.5 \mu\mathrm{L}/\mathrm{min}$$

where $w=0.1 \mathrm{cm}$ and $h=0.015 \mathrm{cm}$ for the fabricated microfluidic chip. At the inlet, a fully developed velocity profile was applied that gave the desired flow rate, and the average exit gauge pressure was set to 0 Pa.

**Fig. 2:**
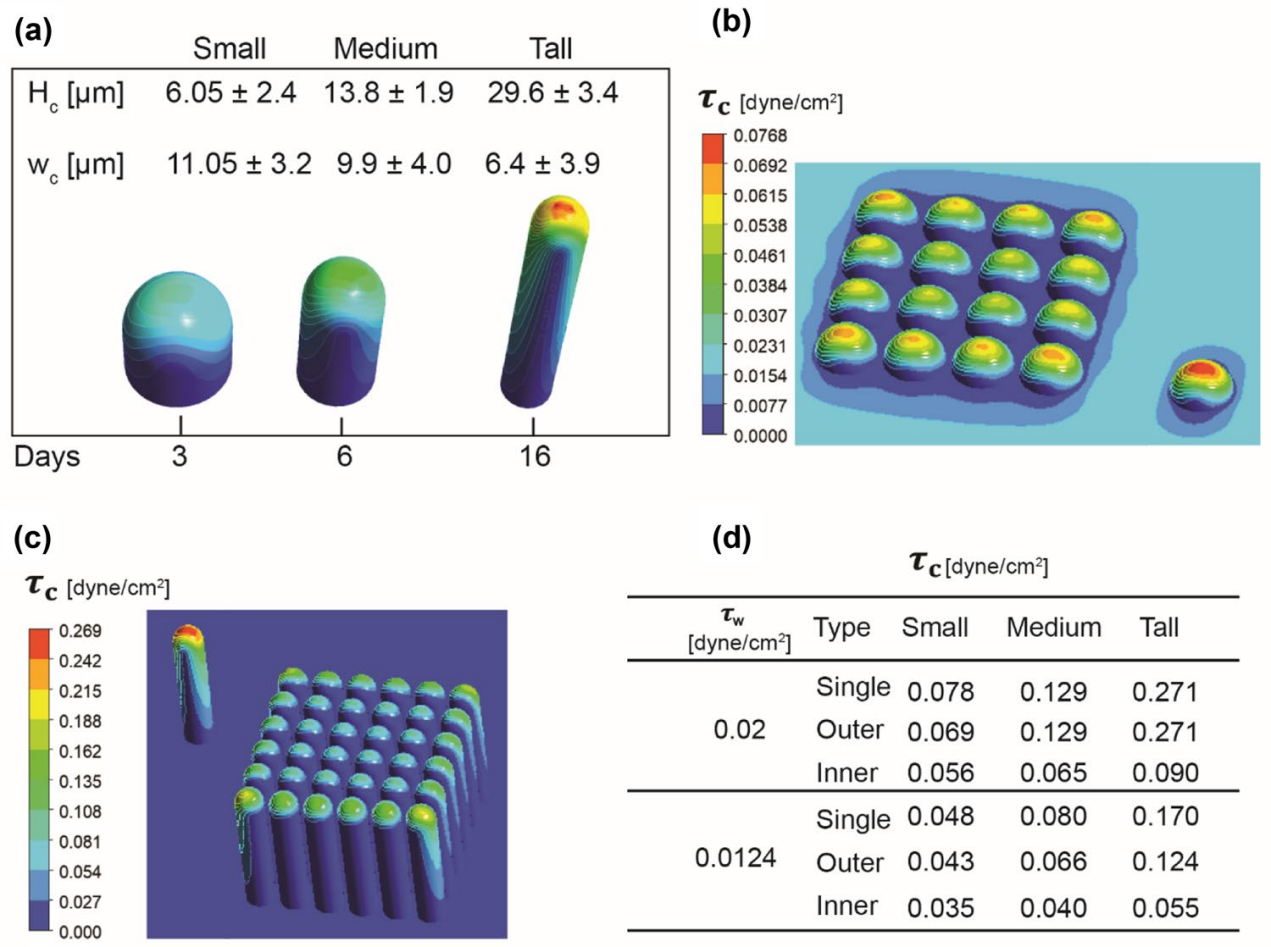
(**a**) Morphologies of cells, small, medium and tall, modelled in Ansys Fluent and based on real Caco-2 cells dimensions, representing typical dimensions respectively at day 3, 6 and 16 of cell growth and observed in standard cell culture (Hidalgo et al. 1989). H_c_ = cell height, w_c_ = cell width, Days = days of culture. (**b**, **c**) Cell shear stress (${\tau }_{c}$) as predicted by CFD simulations for (**b**) small and (**c**) tall cell morphologies in Ansys Fluent. (**d**) Summary of cell shear stress predictions from CFD simulations at ${\tau }_{w}$ = 0.02 dyne/cm^2^ corresponding to a flow rate of 29 µl/hr, and at ${\tau }_{w}$ = 0.0124 dyne/cm^2^ corresponding to a flow rate of 18 µl/hr

Previously reported Caco-2 cells dimensions (Hidalgo et al. 1989) were used as a reference for the setup in Ansys Fluent. Cell height (H_c_) and width (w_c_) correspond to those observed *in vitro* at different time points during 21 days of standard cell culture. Based on these dimensions, three types of cells geometries and arrangements were built in Ansys Fluent for the CFD simulations and are defined as small, medium, and tall, respectively, to indicate their growth. A schematic representation of the cells with dimensions is shown in Fig. 2(a).

### Experimental setup and design

Following cell adhesion, the chips were connected to a peristaltic pump (Longer BT100-2 J, 10 rolls) equipped with micro-flow rate tubes (Choice Analytical SC0189) previously sterilized by running 70% ethanol. The setup included sterile glass reservoirs for the fresh media connected to the inlets of the chips and spent media collected from the outlets. The setup was placed in a cell culture incubator as shown in Fig. 1(e) and all experiments were performed at 37 °C and 5% CO_2_. For testing the effect of shear stress during cell polarization, the flow rate was kept either constant across the experiment at 29 µl/hr, or the flow was decreased to 18 µl/hr between day 5 and day 8. The experimental design is summarized in Fig. 1(d). Cells grown in static conditions in the gut-on-a-chip were cultured at the same cell density and same ECM coating, however, the media was manually replaced daily.

### ddPCR analysis

Gene expression analysis was performed via droplet digital polymerase chain reaction (ddPCR). First, Caco-2 cells were washed once with PBS Rnase-free (Thermo Fisher Scientific, Cat No. AM9624) and then directly lysed on-chip chips or plates. For RNA extraction the RNeasy Plus mini kit (Qiagen, Cat No. 74134) was used as per standard protocol. RNA quality check was performed for all samples with a 4200 TapeStation System by Agilent Technologies (G2991AA). Then, RNA samples were prepared for cDNA synthesis following the protocol by Agilent Technologies for the AffinityScript QPCR cDNA synthesis kit (Cat. No. 600559). All primers were purchased from Sigma as Easy Oligos and the forward (FW) and reverse (RV) sequences are listed next for each gene. For GAPDH FW: GGA GTC CAC TGG CGT CTT CAC, RV: GAG GCA TTG CTG ATG ATC TTG AGG; for ACTB FW: CTG GAA CGG TGA AGG TGA CA, RV: AAG GGA CTT CCT GTA ACA ATG CA; for SDHA FW: TGG GAA CAA GAG GGC ATC TG, RV: CCA CCA CTG CAT CAA ATT CAT G; for HPRT1 FW: TGA CAC TGG CAA AAC AAT GCA, RV: GGT CCT TTT CAC CAG CAA GCT; for ALP1 FW: TGC AGT ACG AGC TGA ACA GGA ACA, RV: TTC ATG GTG CCC GTG GTC AAT; for VIL1 FW: GCT GCT CTA CAC CTA CCT CAT C RV: TTCTGGTCCAGGATGACGGCTT; for TJP1 FW: AGG GGC AGT GGT GGT TTT CTG TTC TTT C; RV: GCA GAG GTC AAA GTT CAA GGC TCA AGA GG; for CDH1 FW: TTC CTC CCA ATA CAT CTC CCT TCA CAG CAG RV: CGA AGA AAC AGC AAG AGC AGC AGA ATC AG; for MUC-2 FW: GTC CGT CTC CAA CAT CAC CT, RV: GCT GGC TGG TTT TCT CCT CT; for MUC-5AC FW: CGA CCT GTG CTG TGT ACC AT, RV: CCA CCT CGG TGT AGC TGA A.

### Immunofluorescence (IF) staining

For immunofluorescence staining, cells were washed with PBS and fixed with 4% PFA in PBS for 15 min. After a subsequent wash with 0.5% Triton X-100 in PBS, a 5% goat serum solution in PBS was used for blocking and was incubated for one hour at room temperature. The following monoclonal primary antibodies were incubated overnight at 4 °C: rabbit anti-Mucin-2 (1:50; Novus biologicals NBP2-66,961), mouse anti-ZO-1 (1:100; ThermoFisher 33–9100), rabbit anti-villin (1:100; Abcam ab130751). In the following staining step, the secondary antibodies were incubated for one hour at room temperature, either combined or not, depending on the staining: goat anti-mouse AF594 (1:200; ThermoFisher, A-11032), goat anti-mouse AF488 (1:200; ThermoFisher, A32723), goat anti-rabbit AF488 (1:200; ThermoFisher, A-11034), goat anti-rabbit AF568 (1:200; ThermoFisher, A-11036). In some cases, staining with conjugated Phalloidin-iFluor 488 antibody was also performed (1:500; Abcam ab176753). Finally, nuclei were stained with 4′,6-diamidino-2-phenylindole (DAPI) (1:1000; Sigma D8417) incubated for 5 min at room temperature. Cells were imaged with an Olympus FluoView FV3000 confocal microscope.

Analysis of cell heights was performed with the ImageJ software after setting the scale available from the confocal microscopy. A total of 309 projections were analyzed and around 820 height individual measurements of monolayers per condition were taken.

### Scanning electron microscopy (SEM)

Cells were prepared for SEM analysis following the standard procedure. Briefly, after culturing in the gut-on-a-chip devices, cells were first washed with phosphate buffer saline (PBS) and then fixed on-chip with 4% paraformaldehyde (PFA) in PBS for 15 min. Cells were further fixed with 2.5% glutaraldehyde in PBS for 30 min at room temperature. Samples were further washed with PBS and fixation with 1% osmium tetroxide in PBS was performed at room temperature for one hour. Following this, dehydration series were performed with 30, 50, 70, 90, 95 and 100% ethanol in Milli-Q water. Finally, the samples were dried with hexamethyldisilazane (ProSciTech Australia, C108), for 2 min. The samples were finally placed in a vacuum desiccator and left to dry overnight. On the following day, samples were sputter-coated with 15 nm of gold (CCU-010 sputter coater, Safematic, Switzerland) and SEM images were taken with a Zeiss Sigma Gemini microscope.

### Statistical analysis

One-way ANOVA with Tukey’s multiple comparisons test was used for the statistical analysis of gene expression and cell heights measures, on GraphPad Prism version 8.4.3 for Windows, GraphPad Software, San Diego, California USA.

## Results and discussion

### Computational modelling to determine flow rate and cell shear stress

Caco-2 cells change their height and morphology resembling the columnar shape of the intestinal villi, with typical dimensions shown in Fig. 2(a). This morphology is different from the one adopted in the *bulge model* theory, in which cells are considered as hemispherical bodies inside a microchannel, referring to the example of leukocytes adhering to the endothelial vessels surface (Gaver III et al. 1998). The *bulge model* theory (Gaver III et al. 1998), is still accepted today to describe the shear stress experienced by cells in microfluidic channels. Given this important difference with the case of villi-like morphology of intestinal cells, we have run CFD simulations to investigate the shear stress experienced by Caco-2 cells during three different morphological changes towards their polarization. Furthermore, we examined the relationship between shear stress at the wall ${\tau }_{w}$ and cell shear stress ${\tau }_{c}$. The ratio between the two values has been derived after the *bulge model* (Gaver III et al. 1998), and describe also by others more recently (Zhang et al. 2014). As a result, it is often accepted that ${\tau }_{c}$ is three times ${\tau }_{w}$. Nevertheless, we evaluated their values and ratio as a function of the volumetric flow rate $Q$. Additionally, as the bulge model refers specifically to single cells, we ran CFD simulations for both single cells and for arrays of cells inside the microchannel at the three different morphologies defined in Fig. 2(a) (small, medium, tall) and cell organizations represented in Fig. 2(b) and (c). We investigated whether the latter arrays of cells could be a better representation of the cell culture *in vitro* when compared with the scenario of a single cell previously considered in the *bulge model*. Therefore, we also observed the shear stress experienced at the front of the arrays and here named as “outer”, and within the arrays “inner”.

The constant flow rate value Q = 29 µl/hr, previously obtained from Eq. (3) and corresponding to a value of shear stress at the walls of 0.02 dyne/cm^2^, was used to run the simulations at the three different morphologies and for the case of single and arrays of cells. Results for the predicted cell shear stress values are shown in Fig. 2(d), where it can be observed that the ratio between cell and wall shear stress is not always three as described by the theoretical *bulge model*. Specifically, we found that the model provides a good approximation only in the case of small cells, which is not surprising given that Gaver and Kute referred to cells approximated as “bulges”. However, for the case of tall cells, which represents polarized Caco-2 cells, the ratio is above four times the shear stress at the wall. These results suggest that Caco-2 cells may be able to experience shear stress values higher than what was previously hypothesized by the *bulge model* (Gaver III et al. 1998).

Finally, we investigated the case of a decreased flow rate, with the same simulations run at a reduced Q = 18 µl/hr corresponding to a ${\tau }_{w}$ of 0.0124 dyne/cm^2^. As a result of the decreased flow rate, the cells experience lower shear stress, with ratios between cell and wall shear stress $({\tau }_{c} /{\tau }_{w})$ around three only for the case of small and medium cells, and above four for the case of polarized (tall) cells. As predicted by the CFD simulations and reported in Fig. 2(d), the cell shear stress experienced by taller cells at the decreased flow rate condition was around 0.05 dyne/cm^2^, which is the same experienced by small cells (approximated as bulges) at their early stage of cell growth.

### Early shear stress produces domes and microvilli formation

The conditions predicted from CFD simulations were examined in the *in vitro* gut-on-a-chip designed in this study. Three different flow conditions were compared: (1) a constant flow rate of 29 µl/hr throughout cell growth within 8 days in the microfluidic channel; (2) a decreased flow rate of 18 µl/hr from day five for another 3 days after domes formation; and (3) cells cultured under static conditions (zero flow) with daily media changes.

**Fig. 3:**
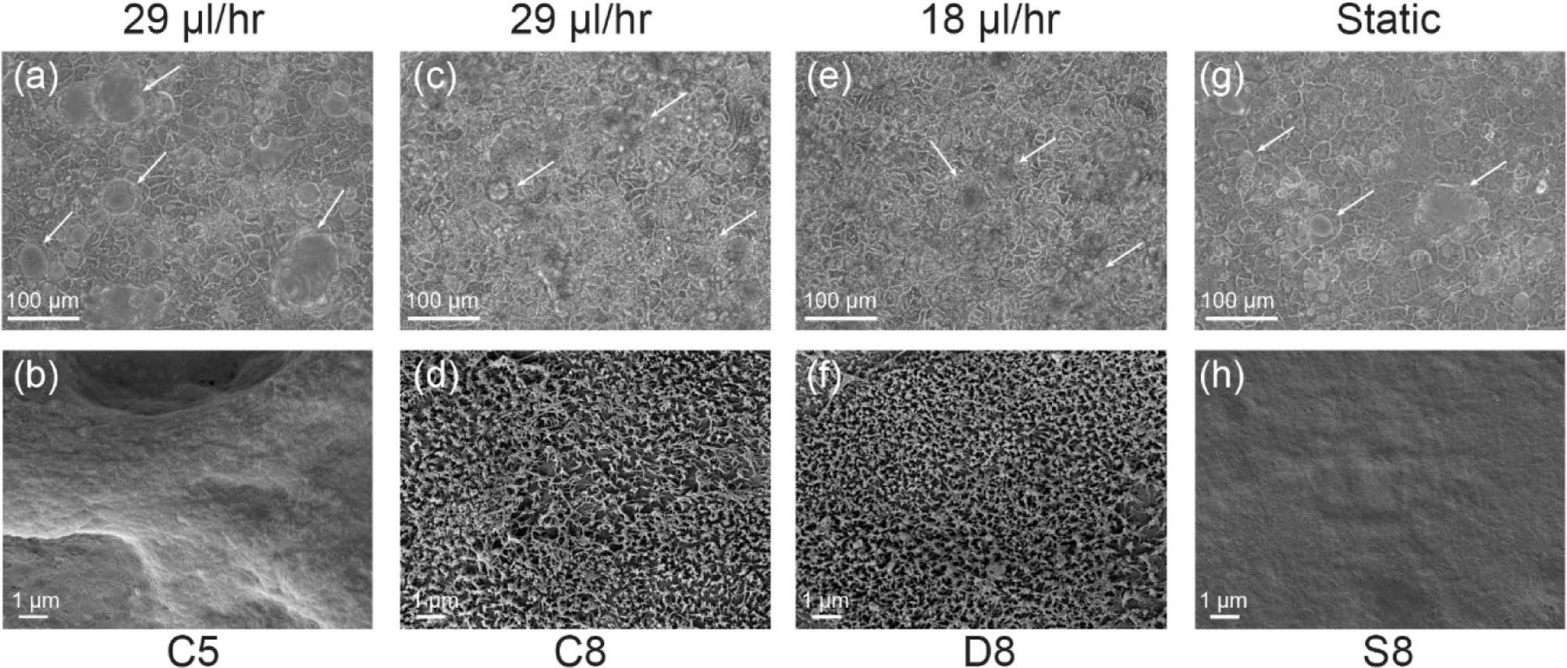
Phase-contrast grayscale photographs (**a**, **c**, **e**, **g**) and SEM micrographs (**b**, **d**, **f**, **h**) of Caco-2 cells under microfluidic conditions. (**a**, **b**) Cells under microfluidic conditions at constant flow (C5) for three days already developed the domes structures (white arrows) and started forming crypt-villi structures. (**c**-**f**) Fully polarized cells were formed at both conditions of constant (C8) and decreased (D8) flow rate with smaller dimensions, domes (**c**, **e**) and fully covered by dense microvilli (**d**, **f**). (**g**-**h**) Cells grown on-chip in static condition (S8) started forming fewer domes by day 8 (**g**) while microvilli were absent (**h**)

At first, cells adhered to the channels overnight (Fig. S1 supplementary information) and on day 2 the gut-on-a-chip devices were connected to the peristaltic pump. As shown in Fig. 3(a), a constant flow rate (C5) of 29 µL/hr for the three first days promoted the formation of domes (white arrows) on Caco-2 cells, which was an indication of enterocytes polarization. However, no microvilli were observed at this condition, while the cells seemed to start forming crypt-villi structures, as shown in the SEM micrograph in Fig. 3(b). The results from both phase-contrast and SEM microscopy of cells grown for three days under microfluidic conditions, confirm that cells are not fully polarized at this stage. For this reason, we chose day 5 as a good time point to investigate the effects of flow variation. Conversely, on the same day 5, no domes formation and microvilli development were observed for cells grown under static conditions in the gut-on-a-chip (Fig. S2 supplementary information).

The results of microscopy analyses, as shown in Fig. 3(c) and (e), illuminated that after eight days dense domes were observed for both flow rates, constant (C8) or decreased (D8), compared to the static conditions (S8) on the same day. Importantly, we highlight that reducing the flow rate after day 5 of the experimental runs, had no significant impact on cell differentiation, when compared to the case of constant flow. Furthermore, it was possible to observe a different arrangement of the cell monolayer, with smaller cell dimensions and organized junctions when compared to cells in static conditions. The smaller dimensions suggest that the cells have fully polarized and this is in line with the observations by Hidalgo et al. (Hidalgo et al. 1989). In static conditions (S8) the cells seem to be at an earlier stage of their growth, with the microscopy photos in Fig. 3(g) indicating that their polarization is not completed after 8 days in the gut-on-a-chip.

Fully developed and dense microvilli covered polarized cells grown at both flow rate conditions, as observed in the SEM micrographs in Fig. 3(d) and (f). Finally, no microvilli were observed in the case of cells grown inside the chip in static conditions (S8), showing a flat monolayer as observed in Fig. 3(h). This data confirmed that cell polarization and microvilli formation was dependent on the sole effect of cell shear stress (${\tau }_{c}$) rather than any possible effect due to Matrigel coating. In parallel, cells were also grown on glass coverslips as per the standardized method, and SEM micrographs (Fig. S3 supplementary information) showed that microvilli formation was reached only by day 21 of culture. However, microvilli grown under this condition appeared less dense if compared to the previous case of cells grown under microfluidic conditions, indicating that shear stress plays a significant role as a biomechanical cue for cell polarization. Our tests under the effect of shear stress in the range of 0.0124 and 0.02 dyne/cm^2^, confirmed that once cell polarization is initiated, microvilli formation is also faster when compared to the case of standard cell culture when cells polarize within 14 to 23 days of cell culturing after confluence (Hidalgo et al. 1989; Lea 2015). Our findings are in line with other published studies which also show that microfluidic conditions can expedite Caco-2 cells polarization and microvilli formation if compared with the standard cell culture on plates (Kim et al. 2012; Delon et al. 2019).

The formations of domes, tight junctions, and a brush border in the apical position, are an indication of complete polarization of Caco-2 cells which, at this stage of their growth, can recapitulate the enterocytes of the small intestine. The presence of microvilli is one of the most important morphological characteristics of differentiated Caco-2 cells. *In vivo*, villi cover the small intestine and increase the surface area available for nutrients absorption during homeostasis. Compromised villi or microvilli formation are usually indications of intestinal malabsorption, dysbiosis and chronic immune diseases. Hence, microvilli formation on Caco-2 cells grown under the microfluidic conditions is a relevant aspect that turns our gut-on-a-chip model into a platform for *in vitro* testing.

### Genetic markers were not altered by dynamic flow rate

**Fig. 4:**
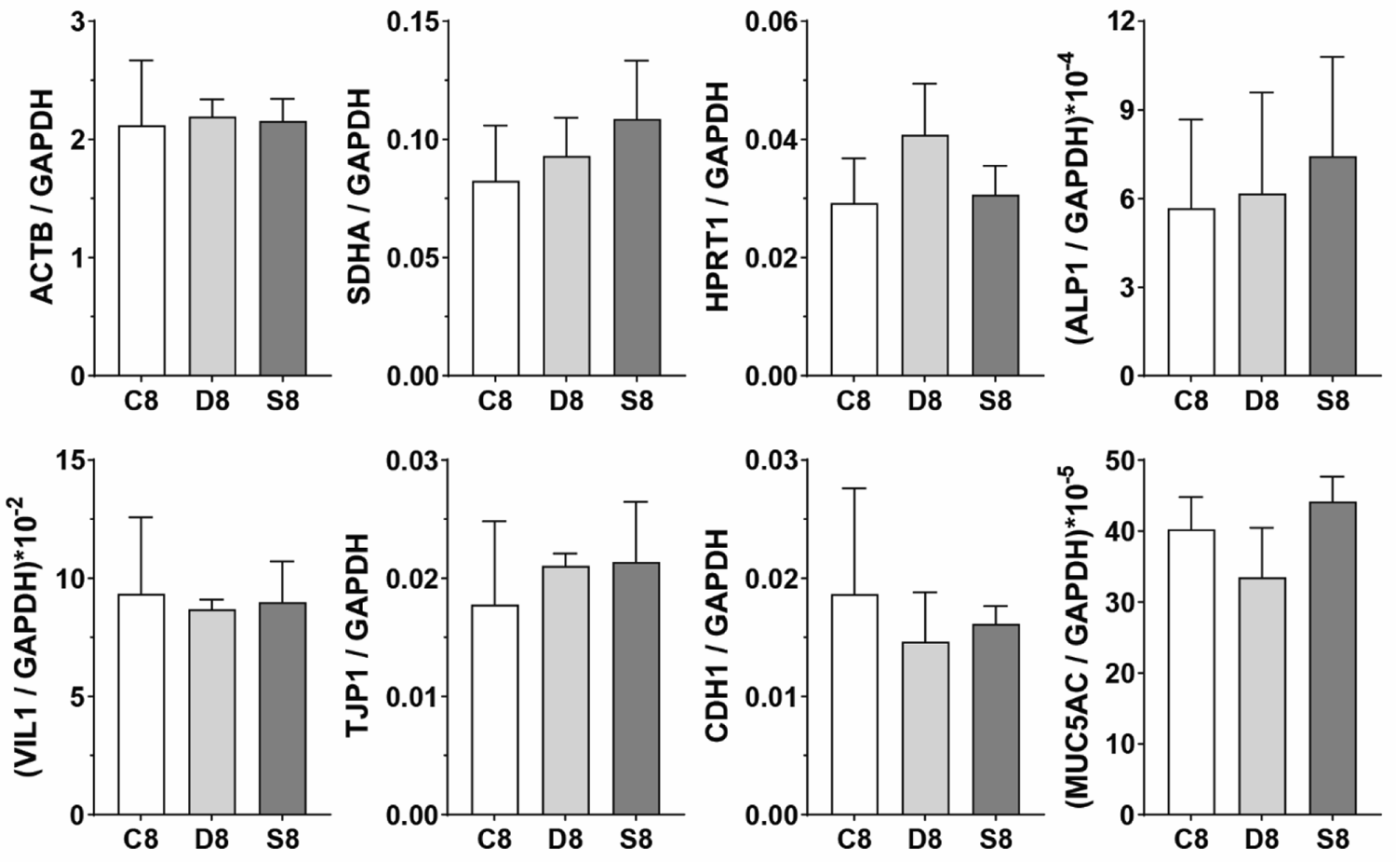
Expression of genes detected via ddPCR analysis as ratios of the means versus the housekeeping gene GAPDH. C8, D8, S8 indicate respectively constant, decreased, and static conditions for samples at day 8 of the experimental runs. Data are presented as mean $\pm$ SD, one-way ANOVA with Tukey’s multiple comparisons test showed no significant difference between conditions ($p>$ 0.05)

We investigated whether genes of interest were differentially expressed depending on the flow conditions used in the gut-on-a-chip device. Results from ddPCR analysis are shown in Fig. 4 and are presented versus GAPDH, which was used as a reference gene. Raw data of the ddPCR analysis are available in Table S1 of the supplementary information. Caco-2 cells do not express high levels of the MUC2 gene, and indeed MUC2 transcript was challenging to detect. We speculate that the high cell shear stress (as predicted by our CFD simulations ${\tau }_{c}$= 0.09 dyne/cm^2^) may impact MUC2 gene expression, however, this would require further investigation and a higher number of biological replicates. Furthermore, low MUC2 expression was still detected at the constant flow rate condition. MUC5AC is reported to be expressed at higher levels (Bu et al. 2011) and was robustly detected under constant and dynamic flow conditions and to a lesser extent under static conditions. Previous studies found that a value of shear stress higher than 0.02 dyne/cm^2^ negatively impact cells morphology and proteins expression (Chi et al. 2015; Delon et al. 2019), arguing against higher flow rates.

ALP1 (IAP), intestinal alkaline phosphatase, and VIL1 respectively indicate cell differentiation and brush borders development and they were expressed in the same range for all samples with variations due to differences between replicates (Table S.1 supplementary information).

Junctions between cells were also the object of our investigation, encoded by the TJP1 gene for tight junctions and the CDH1 gene for *adherens* junctions (E-Cadherin protein). CDH1 gene was expressed slightly at higher levels in the case of constant flow (C8) as if the prolonged shear stress had somehow stimulated this gene responsible for *adherens* junctions between cells. Tight junctions encoded by TJP1 behaved oppositely. However, both CDH1 and TJP1 were expressed in the same ranges for all conditions with no significant up- or downregulations.

### Shear stress influences 3D cell organization and mucin expression

Next, we investigated whether a lower value of shear stress experienced by the cells could potentially impact their morphology and 3D architecture organization. Immunofluorescence (IF) staining was employed to detect the presence of proteins linked to monolayer integrity and cell polarization or illustrative of cell morphology. This included detection of F-actin, ZO-1, villin, and Mucin-2.

**Fig. 5:**
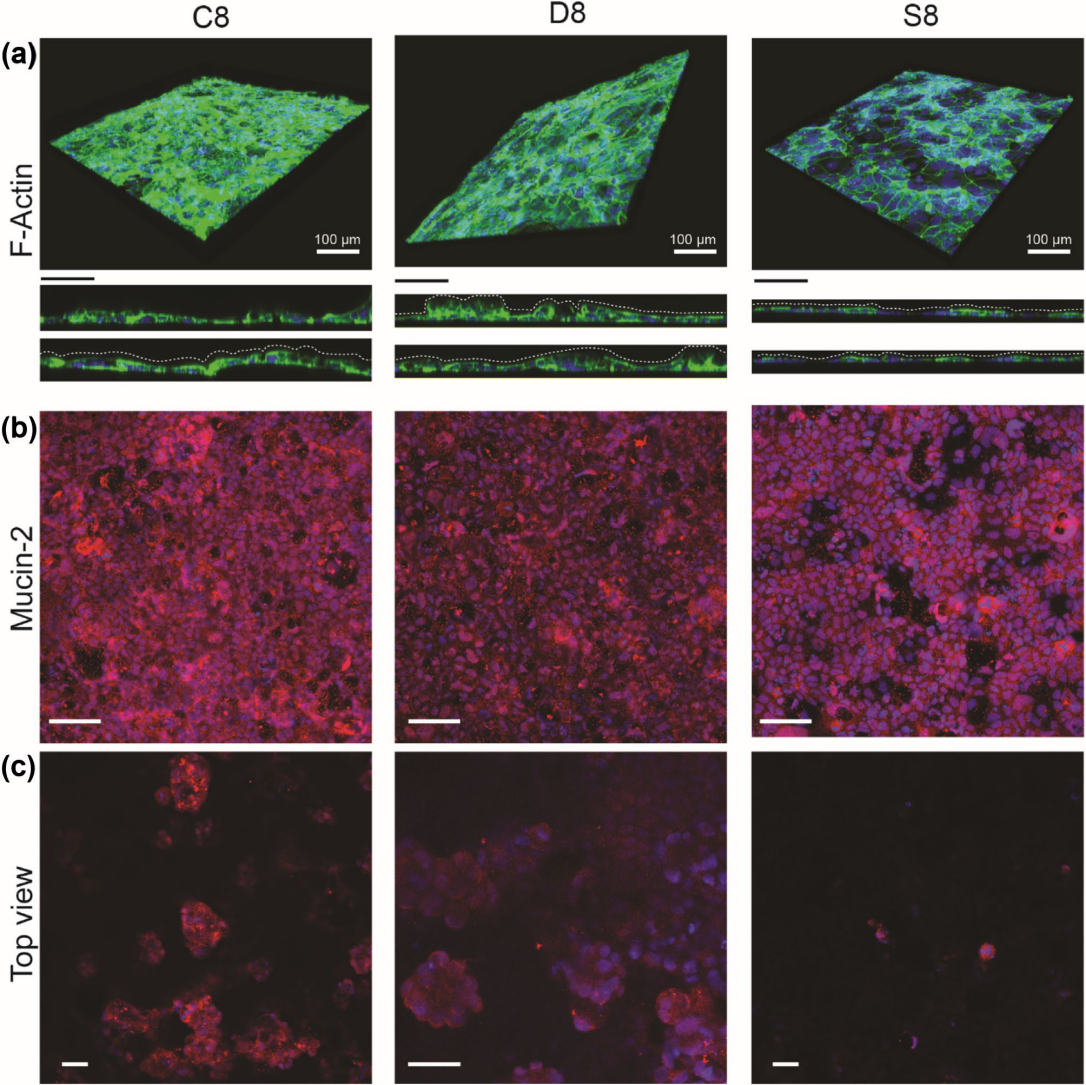
(**a**) Merged volumes photographs of F-Actin (green) and nuclei (blue) staining of Caco-2 cells grown until Day 8. Dashed white lines show the undulating profiles of the monolayers. (**b**) Merged images for nuclei (blue) and Mucin-2 (red) (scale bars 100 µm). (**c**) Top view photographs of the villi tips showing Mucin-2 in the apical position (scale bars 50 µm)

Both conditions of constant (C8) or decreased (D8) shear stress enhanced F-actin formation as shown in Fig. 5(a) when compared with the static conditions (S8) on-chip. F-actin appeared to be distributed at different heights of the sample. 3D volumes and Z-stack projections of the monolayers show undulating profiles of the cell monolayers resembling the *in vivo* morphology of intestinal cells for the case of microfluidic conditions. Other studies in gut-on-a-chip have also demonstrated that polarized Caco-2 cells under microfluidic conditions tend to develop such 3D architectures resembling the crypt-villi organization found *in vivo* (Kim et al. 2012; Pocock et al. 2017; Chi et al. 2015).

**Fig. 6:**
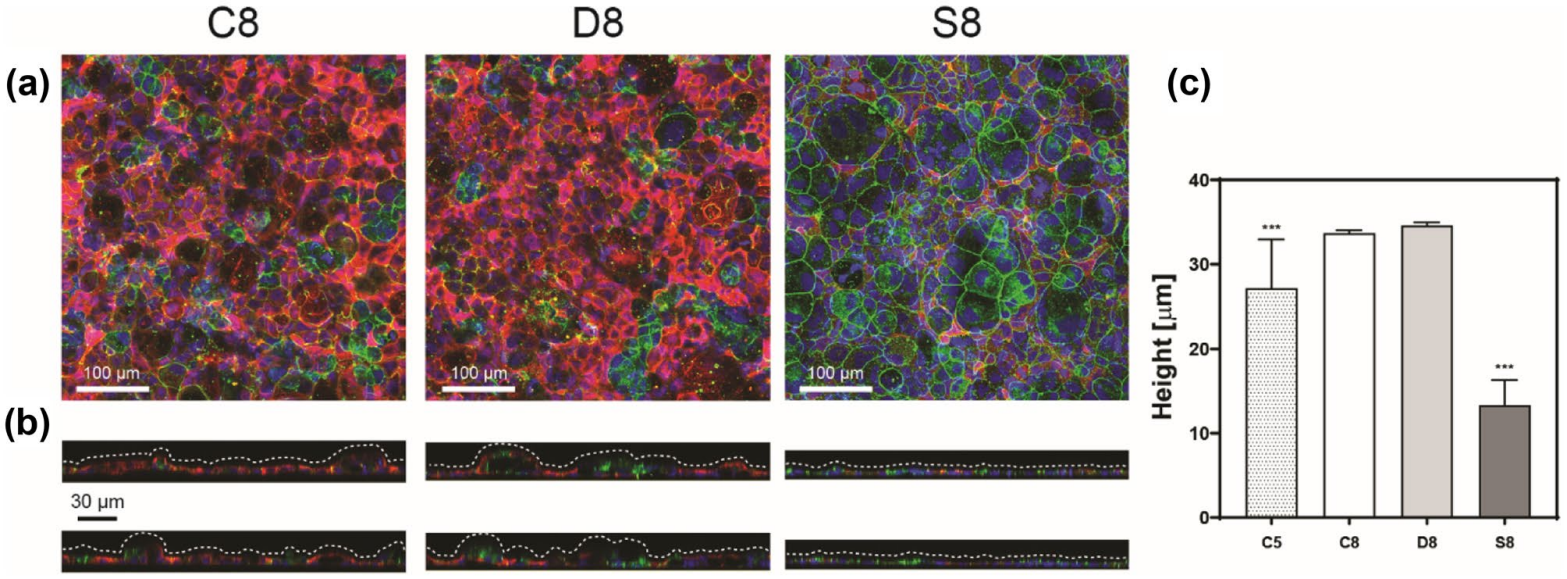
Confocal images for constant (C8) and decreased (D8) flow rate conditions and for static (S8) on day 8. (**a**) Merged photographs for ZO-1 (green), Villin (red), and nuclei (blue). Scale bars represent 100 µm. (**b**) Cell projections for the same staining showing undulating profiles for microfluidic conditions. (**c**) Heights of cell monolayers measured from cell projections (one-way ANOVA with Tukey’s multiple comparisons test, *** $p<$ 0.001). Data are presented as mean $\pm$ SEM

Tight junctions were imaged post IF staining for zonula occludens-1 (ZO-1). Smaller junctions with more uniform dimensions across the monolayer were observed for both constant (C8) and decreased (D8) conditions as shown in Fig. 6. This agrees with the morphology of polarized cells, which possess a smaller diameter and tend to elongate. For the case of no flow (S8), in static conditions, cells developed larger junctions, confirming earlier stages of cell growth resembling “bulges”. The presence of ZO-1 protein indicates the epithelial barrier development and controls the paracellular transport (Lea 2015) hence the formation of tight junctions is relevant in models of the intestine *in vitro*.

Microvilli formation was investigated via IF staining for villin protein. Villin is a marker of Caco-2 cell differentiation and its presence has been previously reported by Kim and others in both crypts and villi regions of Caco-2 cells (Kim and Ingber 2013). The confocal images in Fig. 6(a) and (b) show the presence of villin, confirming brush border formation and 3D organization of the cell monolayer, while it is clear the lack of villin in the case of static conditions on-chip. This agrees with the previous observations of F-actin distribution and undulating profiles in Fig. 5(a), suggesting that a shear stress value of 0.0124 dyne/cm^2^ does not impact cell polarization.

Another important characteristic to consider when developing *in vitro* models of the gut is the presence of a mucus layer, which is naturally present on the intestinal epithelium in homeostatic conditions. We investigated whether the decreased shear stress could potentially influence mucin expression. The immunofluorescence images in Fig. 5(b) show IF staining for Mucin-2. At both flow rate conditions (C8 and D8) in the gut-on-a-chip, the expression of mucin was not impacted. Top view images of the cells in Fig. 5(c), were taken at the maximum Z value for each sample and show Mucin-2 distributed to the villi tips. In contrast, cells grown in static (S8) conditions on-chip did not show any mucin formation or villi tips, in agreement with the missing 3D architecture when no shear stress is applied. Although Mucin-2 was lowly expressed at a gene level in Caco-2 cells, shear stress in microfluidics allowed for robust Mucin-2 expression at the protein level. Several prior gut-on-a-chip studies have investigated the effect of shear stress on Caco-2 cells and reported Mucin-2 expression at the protein level, in microfluidic chips at a shear stress of 0.02 dyne/cm^2^ (Delon et al. 2019; Chi et al. 2015; Kim and Ingber 2013). We demonstrate that Mucin-2 expression is still seen under a decreased shear stress of 0.0124 dyne/cm^2^, as long as cell polarization is initiated at 0.02 dyne/cm^2^.

To further investigate whether the 3D cell architecture could be impacted by the decreased shear stress during their growth, the 3D projections in the XZ and YZ directions of the confocal images were used for quantifying the cell monolayers’ height in Fig. 6(b). The results in Fig. 6(c) were obtained by measuring cell monolayers heights from confocal images taken across different sections of each chip at the different fluidic conditions. By day eight cells grown in static conditions formed a thin monolayer with flat profiles of an average height of 13 µm, which is in line with previously published measures of non-differentiated bulge-like (small) Caco-2 cells (Hidalgo et al. 1989). The case of constant flow on day 5 (C5), in the middle of the experiment, showed more variation across the different samples analyzed, with an average height of 27 µm resembling the case of medium cells geometry previously used for the CFD simulations (Fig. 2(a)). In this case, cells have started polarizing partially after only three days under microfluidic conditions, resulting in a high variation of the cell monolayer’s height. Cells kept under microfluidic conditions for a total of six days developed thicker monolayers. The mean height value of cell monolayers for both flow conditions was 34 µm, underpinning no impact by the decreased flow condition. These data are in line with the dimensions observed by Hidalgo (Hidalgo et al. 1989) and which were previously used in our CFD simulations. Furthermore, cell monolayers’ average height in gut-on-a-chip has been previously reported to be around 20 to 30 µm for the case of fully polarized (tall) cells (Kim et al. 2012; Delon et al. 2019; Wang et al. 2019) under the effect of a constant flow rate and shear stress of 0.02 dyne/cm^2^. Our results confirmed that the cell monolayer architecture and height are not affected by the decreased shear stress of 0.0124 dyne/cm^2^ and are within the expected ranges of mean height for Caco-2 cells.

## Conclusions

Combining in silico CFD simulations and *in vitro* microfluidic culture has given us unique insights into the relationship between cell differentiation and morphology and biomechanical cues such as shear stress. CFD modeling helped to establish an optimal calculated shear stress for culturing Caco-2 cells in a gut-on-a-chip model. The previously reported optimal shear stress of 0.02 dyne/cm^2^ (corresponding to a cell shear stress of 0.09 dyne/ cm^2^ from CFD simulations for tall-polarized cells) is critical to initiate cell polarization at the early stages of culture. Nevertheless, Caco-2 cells grown under conditions of a dynamic flow rate in a microfluidic apparatus were still able to polarize, evidenced by the expression of protein markers of differentiation and development of crypt-villi structures.

Our dynamic flow approach takes into consideration the morphology changes naturally occurring in Caco-2 cells, hence adopting a constant cell shear stress ${(\tau }_{c})$ of about 0.05 dyne/cm^2^ as predicted in our CFD simulations. We initially hypothesised that a decreased flow rate and shear stress might be beneficial for villi formation, however, we found that our approach is comparable to the constant flow rate case and that at least the decreased flow does not hinder villi formation and relevant proteins expression. Therefore, we envision that the decreased flow rate approach developed in this study may be beneficial for the use of gut-on-a-chip for screening the efficacy of active compounds. Pharmaceuticals and biologics can be expensive to test with the standard cell culture approach, with microfluidic models facilitating a considerable reduction of the volumes involved. In this study, we applied this approach to the specific case of Caco-2 cells, but this study raises the potential to study how flow rates and shear stress can affect other cell types with different morphologies. Such studies could be strengthened by comparable approaches incorporating both CFD and practical cell culture experiments.

## Supplementary information

Below is the link to the electronic supplementary material.

Supplementary file1 (PDF 933 KB)

## Data Availability

All data generated or analyzed during this study are included in this published article and its supplementary information. Additional data are available upon reasonable request to the authors.

## References

[CR1] Beaurivage C, Kanapeckaite A, Loomans C, Erdmann KS, Stallen J, Janssen RAJ (2020). Development of a human primary gut-on-a-chip to model inflammatory processes. Sci. Rep..

[CR2] Bu X-D, Li N, Tian X-Q, Huang P-L (2011). Caco-2 and LS174T cell lines provide different models for studying mucin expression in colon cancer. Tissue Cell.

[CR3] Chi M, Yi B, Seunghan Oh, Park D-J, Sung JH, Park S (2015). A microfluidic cell culture device (μFCCD) to culture epithelial cells with physiological and morphological properties that mimic those of the human intestine. Biomed. Microdevice.

[CR4] L.C. Delon, Z. Guo, A. Oszmiana, C.C. Chien, R. Gibson, C. Prestidge, B. Thierry, 'A systematic investigation of the effect of the fluid shear stress on Caco-2 cells towards the optimization of epithelial organ-on-chip models', Biomater. **225**, 119521 (2019) 10.1016/j.biomaterials.2019.119521 31600674

[CR5] Fois CAM, Le TYL, Schindeler A, Naficy S, McClure DD, Read MN, Valtchev P, Khademhosseini A, Dehghani F (2019). Models of the Gut for Analyzing the Impact of Food and Drugs. Adv. Healthcare Mater..

[CR6] Gaver III, Donald P, Kute SM (1998). A theoretical model study of the influence of fluid stresses on a cell adhering to a microchannel wall. Biophys. J ..

[CR7] Gijzen L, Marescotti D, Raineri E, Nicolas A, Lanz HL, Guerrera D, van Vught R, Joore J, Vulto P, Peitsch MC (2020). An Intestine-on-a-chip model of plug-and-play modularity to study inflammatory processes. SLAS TECHNOLOGY: Translating Life Sciences Innovation.

[CR8] Hidalgo IJ, Raub TJ, Borchardt RT (1989). Characterization of the human colon carcinoma cell line (Caco-2) as a model system for intestinal epithelial permeability. Gastroenterology.

[CR9] Hugenholtz F, de Vos WM (2018). Mouse models for human intestinal microbiota research: a critical evaluation. Cell. Mol. Life Sci..

[CR10] Ingber DE (2020). Is it Time for Reviewer 3 to Request Human Organ Chip Experiments Instead of Animal Validation Studies?. Advanced Science.

[CR11] Jalili-Firoozinezhad S, Gazzaniga FS, Calamari EL, Camacho DM, Fadel CW, Bein A, Swenor B, Nestor B, Cronce MJ, Tovaglieri A (2019). A complex human gut microbiome cultured in an anaerobic intestine-on-a-chip. Nature Biomedical Engineering.

[CR12] Kasendra M, Tovaglieri A, Sontheimer-Phelps A, Jalili-Firoozinezhad S, Bein A, Chalkiadaki A, Scholl W, Zhang C, Rickner H, Richmond CA (2018). Development of a primary human Small Intestine-on-a-Chip using biopsy-derived organoids. Sci. Rep..

[CR13] Kim HJ, Huh D, Hamilton G, Ingber DE (2012). Human gut-on-a-chip inhabited by microbial flora that experiences intestinal peristalsis-like motions and flow. Lab Chip.

[CR14] Kim HJ, Ingber DE (2013). Gut-on-a-Chip microenvironment induces human intestinal cells to undergo villus differentiation. Integr. Biol..

[CR15] Kimura H, Yamamoto T, Sakai H, Sakai Y, Fujii T (2008). An integrated microfluidic system for long-term perfusion culture and on-line monitoring of intestinal tissue models. Lab Chip.

[CR16] L. Tor, 'Caco-2 cell line'. The impact of food bioactives on health, 103–11 (2015)

[CR17] Mahler GJ, Esch MB, Glahn RP, Shuler ML (2009). Characterization of a gastrointestinal tract microscale cell culture analog used to predict drug toxicity. Biotechnol. Bioeng..

[CR18] Natoli M, Leoni BD, D’Agnano I, Zucco F, Felsani A (2012). Good Caco-2 cell culture practices. Toxicol. in Vitro.

[CR19] Nguyen TL, Anh S-S, Liston A, Raes J (2015). How informative is the mouse for human gut microbiota research?. Dis. Model. Mech..

[CR20] Pocock K, Delon L, Bala V, Rao S, Priest C, Prestidge C, Thierry B (2017). Intestine-on-a-chip microfluidic model for efficient in vitro screening of oral chemotherapeutic uptake. ACS Biomater. Sci. Eng..

[CR21] C. Poon, 'Measuring the density and viscosity of culture media for optimized computational fluid dynamics analysis of in vitro devices'. bioRxiv, (2020) 10.1016/j.jmbbm.2021.105024 34911025

[CR22] Pound P, Ritskes-Hoitinga M (2018). 'Is it possible to overcome issues of external validity in preclinical animal research? Why Most Animal Models Are Bound to Fail'. J. Transl. Med..

[CR23] Shim K-Y, Lee D, Han J, Nguyen N-T, Park S, Sung JH (2017). Microfluidic gut-on-a-chip with three-dimensional villi structure. Biomed. Microdevice.

[CR24] Den Berg V, Albert CL, Mummery RP, Van der Meer AD (2019). Personalised organs-on-chips: functional testing for precision medicine. Lab Chip.

[CR25] Van Kooten TG, Schakenraad JM, Van der Mei HC, Busscher HJ (1992). Development and use of a parallel-plate flow chamber for studying cellular adhesion to solid surfaces. J. Biomed. Mater. Res..

[CR26] Y. Wang, Z. Shao, W. Zheng, Y. Xie, G. Luo, M. Ding, Q.A. Liang, 'A 3D construct of the intestinal canal with wrinkle morphology on a centrifugation configuring microfluidic chip'. Biofabrication **11**, 045001 (2019) 10.1088/1758-5090/ab21b0 31091514

[CR27] X. Zhang, Huk D.J., Wang Q., Lincoln J. Zhao Y. 'A microfluidic shear device that accommodates parallel high and low stress zones within the same culturing chamber'. Biomicrofluidics **8**, 054106 (2014) 10.1063/1.4894783 PMC4189595 25332743

