# Supplementary material for: Dynamic flow and shear stress as key parameters for intestinal cells morphology and polarization in an organ-on-a-chip model
Source: Biomed Microdevices. 2021 Oct 16;23(4):55. doi: 10.1007/s10544-021-00591-y (PMC8520520; doi:10.1007/s10544-021-00591-y)
Supplement: Supplementary file 1 — Supplementary file1 (PDF 933 KB) [file 10544_2021_591_MOESM1_ESM.pdf]

Supplementary Information

Dynamic flow and shear stress as key parameters for intestinal cells morphology and polarization in an organ-on-a-chip model

Chiara A. M. Fois, Aaron Schindeler, Peter Valtchev, Fariba Dehghani*

School of Chemical and Biomolecular Engineering, The University of Sydney, Sydney, 2006, Australia

Centre for Advanced Food Engineering, The University of Sydney, Sydney, 2006, Australia

*Corresponding author: fariba.dehghani@sydney.edu.au

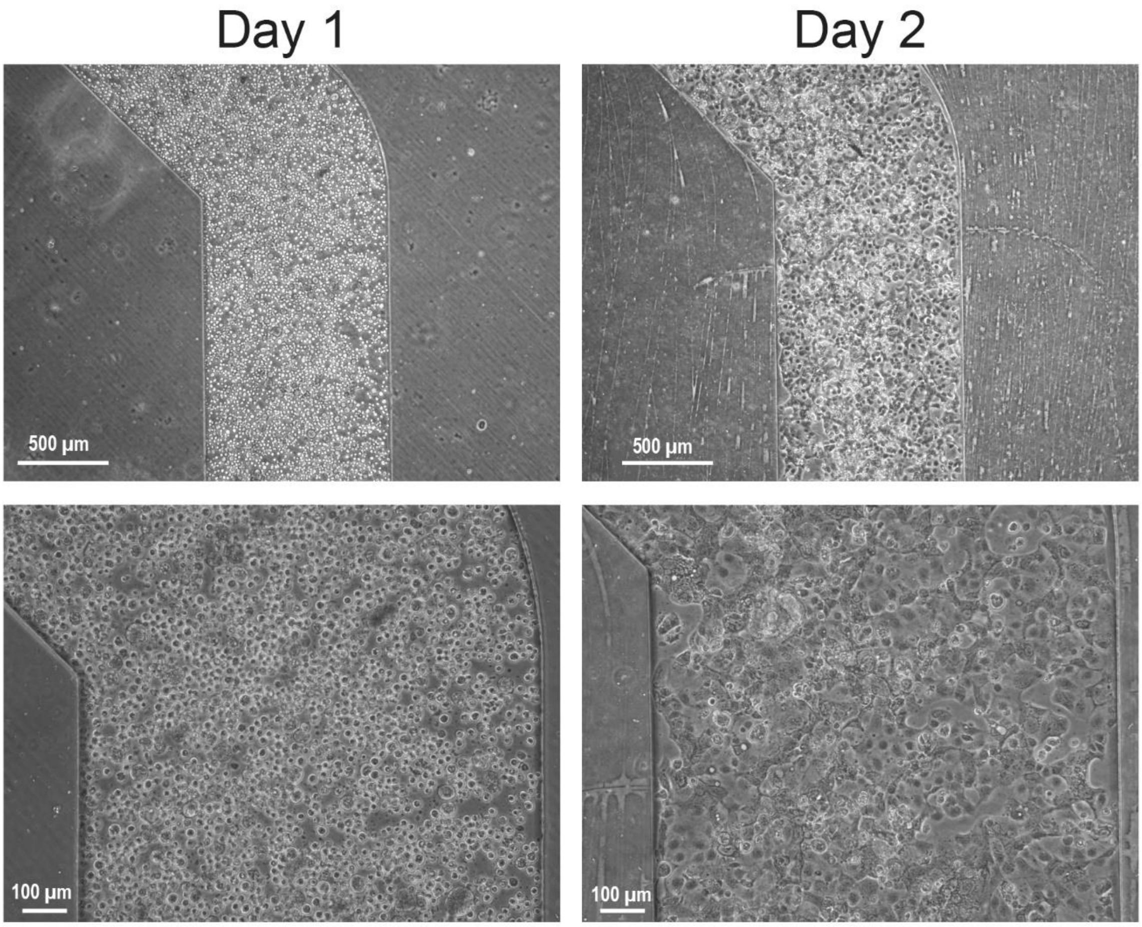

Fig. S1 Phase-contrast grayscale images of Caco-2 cells seeded inside the gut-on-a-chip on Day 1 (seeding day) and Day 2, 24 hours post-seeding.

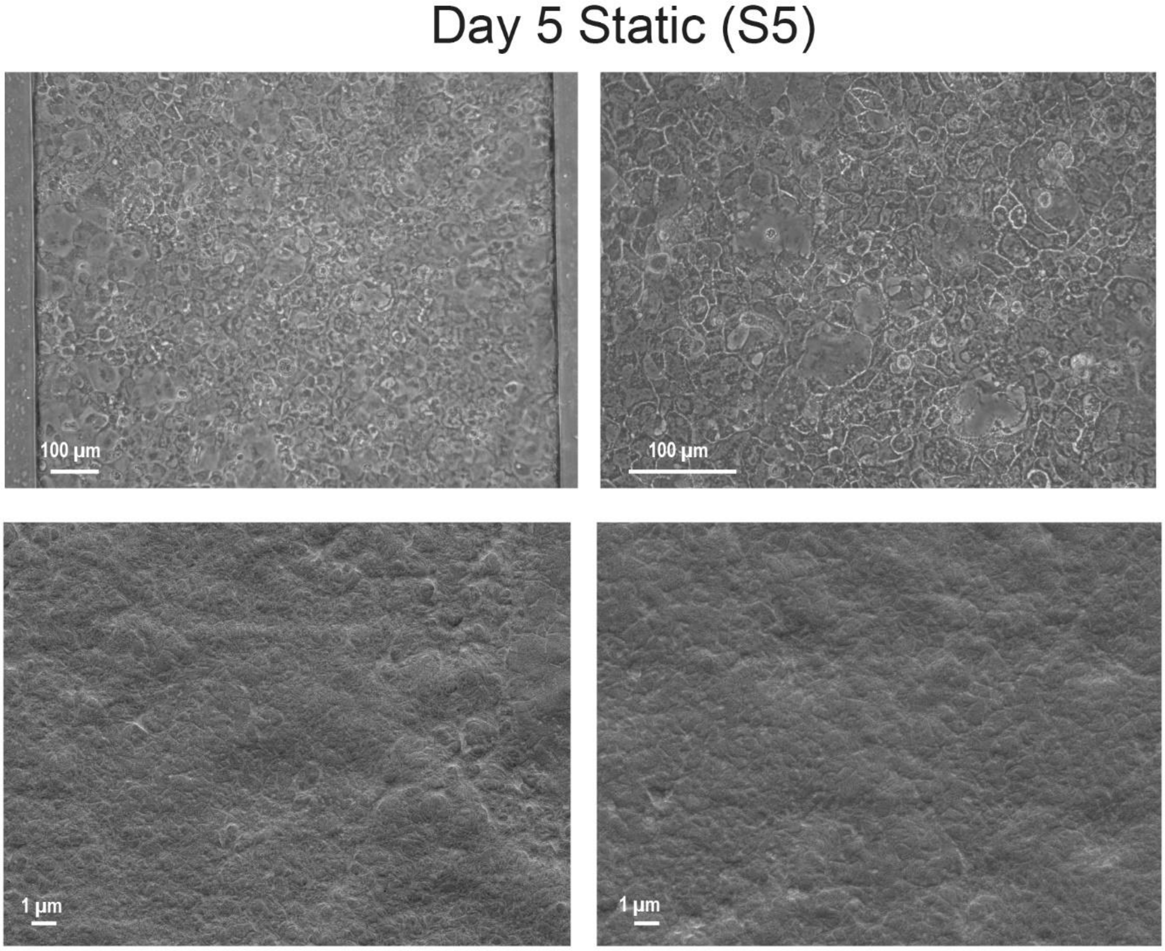

Fig. S2 Phase-contrast grayscale images (top row) and SEM micrographs (bottom row) of Caco-2 cells grown in the gut-on-a-chip for 5 days under static conditions (no flow). No domes formation or microvilli were observed at this condition.

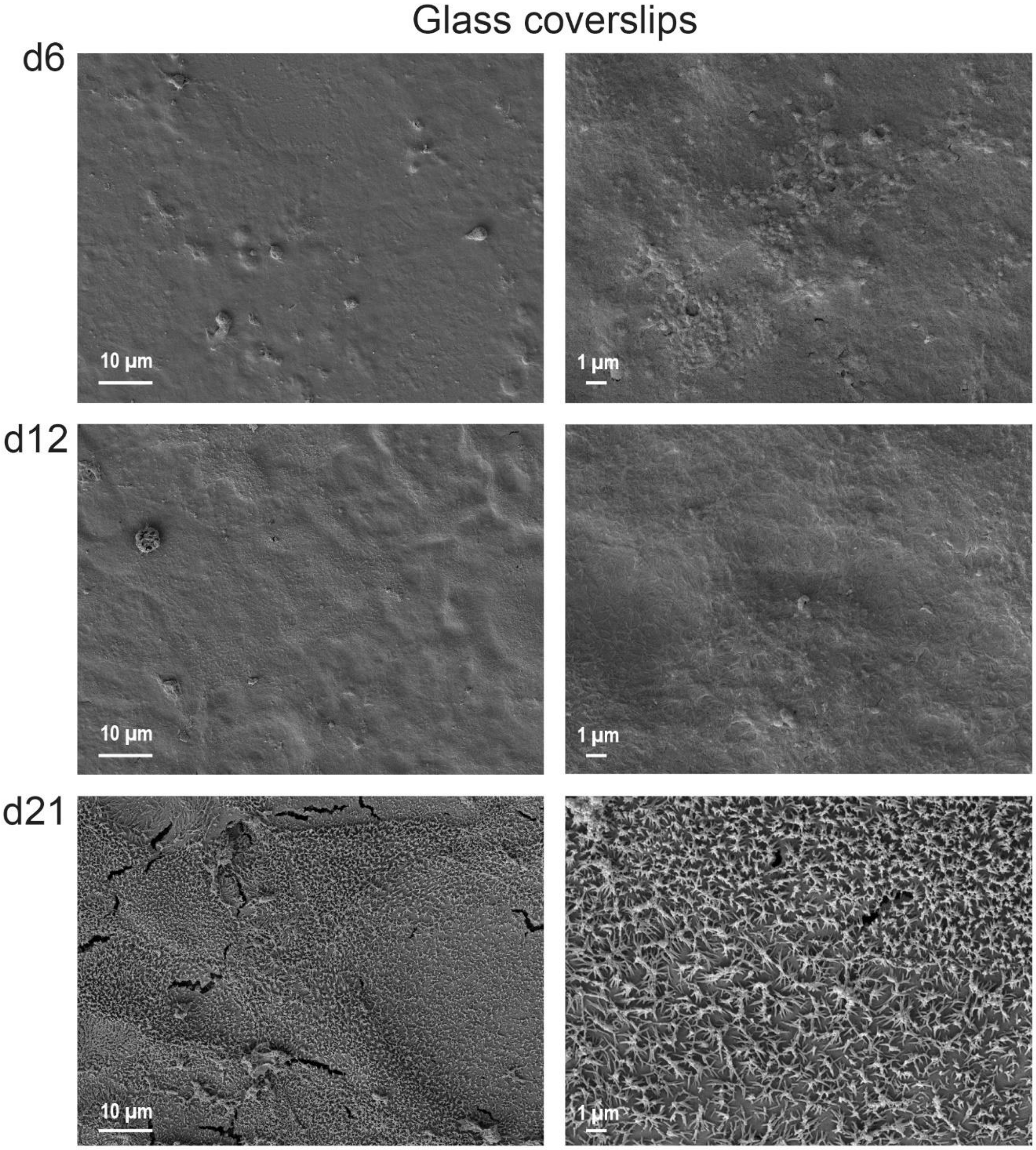

Fig. S3 SEM micrographs of Caco-2 cells grown in static conditions on glass coverslips coated with Matrigel for 6 days (d6), 12 days (d12) and 21 days (d21). Microvilli on Caco-2 cells are visible only after 21 days under standard cell culture conditions.

Table S1 Raw data for ddPCR genes expression analysis for the investigation of flow variation effects on Caco-2 cells. All samples at day 8 of the experimental runs. C8 = constant, D8 = decreased flow rate, S8 = static conditions on-chip. Replicate = biological replicate sample number from independent experiments.

**Conditions**	**C8**			**D8**		**S8**		
**Replicate**	**1**	**2**	**3**	**1**	**2**	**1**	**2**	**3**
**Genes**	**Concentration (copies/µl)**							
GAPDH	2991	1526	3480	2350	2073	2240	1487	1844
ACTB	6330	3980	6660	4630	5080	4380	3690	3940
SDHA	258	147.9	254	231	180.5	251	160.1	194.6
HPRT1	86.2	55	92.8	76.7	103.7	67.3	50.1	53.3
ALP1 (IAP)	1.54	0.7	2.3	1.9	0.83	2.1	1.04	1
VIL1	303	149.6	295	186	198.6	201	137.5	162.6
TJP1	50.2	27.3	64.6	48.2	44.9	50.5	32.8	35.8
E-Cadherin	58.4	22.8	68.1	25.9	38.9	28.5	28.3	33.2
MUC-2	0.05	0.05	0	0	0	0	0	0
MUC-5A	1.2	0.96	1.06	0.63	0.85	0.88	0.75	0.83
